# Identifying the sociodemographic and work-related factors related to workers’ daily physical activity using a decision tree approach

**DOI:** 10.1186/s12889-023-16747-9

**Published:** 2023-09-23

**Authors:** Aviroop Biswas, Cynthia Chen, Kathleen G. Dobson, Stephanie A. Prince, Faraz Vahid Shahidi, Peter M. Smith, Daniel Fuller

**Affiliations:** 1 Institute for Work & Health, 400 University Avenue, Suite 1800, Toronto, ON M5G1S5 Canada; 2 Dalla Lana School of Public Health, University of Toronto, Toronto, ON Canada; 3 Centre for Surveillance and Applied Research, Public Health Agency of Canada, Ottawa, ON Canada; 4 School of Epidemiology and Public Health, University of Ottawa, Ottawa, ON Canada; 5 Department of Epidemiology and Preventive Medicine, Monash University, VIC Melbourne, Australia; 6 Department of Community Health and Epidemiology, University of Saskatchewan, Saskatoon, SK Canada

**Keywords:** Physical activity, Machine learning, Workplace, Epidemiology, Decision trees, Exercise

## Abstract

**Background:**

The social and behavioural factors related to physical activity among adults are well known. Despite the overlapping nature of these factors, few studies have examined how multiple predictors of physical activity interact. This study aimed to identify the relative importance of multiple interacting sociodemographic and work-related factors associated with the daily physical activity patterns of a population-based sample of workers.

**Methods:**

Sociodemographic, work, screen time, and health variables were obtained from five, repeated cross-sectional cohorts of workers from the Canadian Health Measures Survey (2007 to 2017). Classification and Regression Tree (CART) modelling was used to identify the discriminators associated with six daily physical activity patterns. The performance of the CART approach was compared to a stepwise multinomial logistic regression model.

**Results:**

Among the 8,909 workers analysed, the most important CART discriminators of daily physical activity patterns were age, job skill, and physical strength requirements of the job. Other important factors included participants’ sex, educational attainment, fruit/vegetable intake, industry, work hours, marital status, having a child living at home, computer time, and household income. The CART tree had moderate classification accuracy and performed marginally better than the stepwise multinomial logistic regression model.

**Conclusion:**

Age and work-related factors–particularly job skill, and physical strength requirements at work–appeared as the most important factors related to physical activity attainment, and differed based on sex, work hours, and industry. Delineating the hierarchy of factors associated with daily physical activity may assist in targeting preventive strategies aimed at promoting physical activity in workers.

**Supplementary Information:**

The online version contains supplementary material available at 10.1186/s12889-023-16747-9.

## Background

Compelling dose–response evidence from observational studies indicates that modest amounts of physical activity (PA) can protect against several chronic conditions (e.g., diabetes, cardiovascular disease, cancer) and premature all-cause mortality among the working-age population, while additional health benefits can be achieved with higher levels of PA [1, 2]. It is increasingly recognised that PA of any type and any duration is beneficial to health and this has led to an increased number of studies examining the health effects of PA of different intensities and durations that people accumulate in their daily lives [3, 4]. As such, understanding the factors associated with daily PA behaviours is essential to the development and improvement of public health interventions by identifying population groups requiring PA promotion to improve health outcomes and quality of life.

Previous research has identified multiple individual, social, and environmental factors associated with daily PA among adults [5–7]. While these studies have generated important insights, they have generally employed standard regression techniques which have been criticized for lacking the flexibility to consider complex interactions and nonlinear associations [8]. Data-driven machine learning techniques offer more flexible modelling of potential correlate-outcome relationships [8]. Of the various machine learning approaches, decision trees have been used to identify multiple factors associated with mental health service use, obesity, smoking studies, and diabetes [9–12]. Decision trees can identify factors most relevant for predicting an outcome of interest and are able to detect and characterize nonlinear associations. Their outputs are easily interpretable in the form of a tree-like flowchart and the approach is capable of classifying individuals that are mutually exclusive from each other according to a hierarchy of factors, can detect multiple interactions without a priori decisions, and does not have the sample size limitations for statistical power to detect effects as regression does [13]. For all these reasons, decision tree methods may offer unique advantages over conventional regression techniques employed in the social and behavioural health sciences.

A small number of studies have used decision tree approaches to examine the factors associated with PA behaviours. For example, Farrahi et al. developed a data-driven decision tree model to explore the relative importance of 36 different factors as predictors of PA profiles among adult participants in the Northern Finland Birth Control Cohort 1966 study [14]. Paxton et al. used similar methods to examine compliance with PA guidelines in African American breast cancer survivors [15], while Lakerveld et. al. examined the hierarchy of sociodemographic correlates associated with high and low sitting time and PA groups [16]. Along with identifying physical health, psychological factors, socioeconomic factors, and the built environment as important factors related to PA attainment, occupation type was highlighted across these studies as an important discriminator of PA attainment [14–16]. However, these studies did not explore the contributions of specific work-related factors (e.g., work stress, work hours, physical demands of work) as potentially associated with PA. Furthermore, previous studies did not examine factors associated with multiple possibilities of daily PA patterns beyond a simplistic, binary outcome of whether weekly PA recommendations were met. This represents a substantial research gap as workers constitute most of the working-age adult population and work-related factors (e.g., physical, and psychosocial job demands) have been independently associated with differences in daily PA [17–19]. Understanding the interrelationships between work and non-work correlates with daily PA patterns can inform targeted intervention approaches that are relevant to large segments of the adult working population.

This study used a decision tree machine learning approach to examine the hierarchy of interrelated factors associated with daily PA behaviour patterns in a population-based sample of Canadian workers. The performance of the decision tree approach was compared to a conventional regression approach.

## Methods

### Participants

The study examined cross-sectional data from the Canadian Health Measures Survey (CHMS), which has a sampling frame representative of > 96% of the Canadian population. Data was collected every two years from a sample of approximately 5,000 Canadians aged 6 to 79 years [20]. The CHMS includes self-reported demographics and health data, accelerometer-measured continuous PA data and clinically measured cardiometabolic markers. Details on the survey design and data collection methods have been described previously [20]. Briefly, the CHMS was designed to produce national-level estimates representative of the Canadian population and its ten provinces and three territories. As well as an in-person household questionnaire to gather socio-demographic, health, and lifestyle information, the CHMS involved participants visiting to a mobile examination center (MEC) for direct physical measures. Respondents who were unable to visit the MEC could opt to have their direct measures taken at home.

Five cycles of cross-sectional CHMS data (2007 to 2017) from ~ 11,250 respondents (49% female) were combined. Participants were included if they were 18 years of age or older, reported having a job, and were at work in the week prior to responding to the survey. Pregnant women were excluded from the sample. A total of 10,582 participants met the inclusion criteria. Among them, 8,909 participants had valid accelerometer data; activity data for 10 h per day and for at least 4 days (participants were told to wear accelerometers on their waist for a week except when sleeping, swimming, or bathing). The University of Toronto’s Health Sciences Research Ethics Board (REB # 40802) approved the study protocol.

### Measures

#### Outcome: Daily physical activity (PA) patterns

**Fig. 1:**
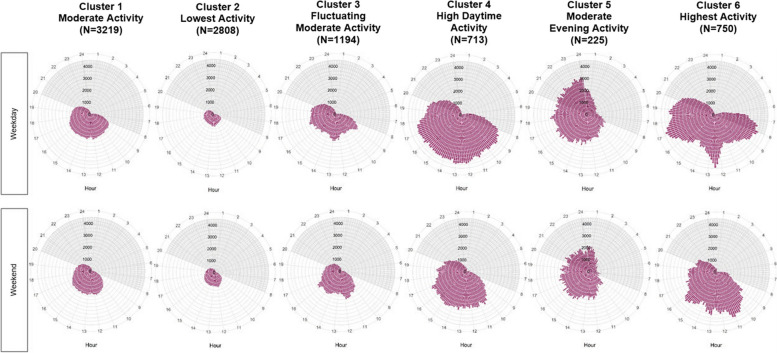
Daily physical activity patterns of working participants (*n* = 8,909)*. *Plots show accelerometer activity counts averaged every 10 min. Weekday and weekend counts shown separately. Dark shade denotes nighttime hours and light shade denotes daytime hours

This study used continuous activity data collected using a waist-worn accelerometer to identify daily PA patterns using hierarchical cluster analysis. PA was grouped into six distinct daily patterns defined in a previous study [21]. The six PA patterns previously identified were labelled as: “lowest activity” (*N* = 2,808), “moderate consistent activity” (*N* = 3,219), “fluctuating moderate activity” (*N* = 1,194), “high daytime activity” (*N* = 713), “moderate evening activity” (*N* = 225), and “highest activity” (*N* = 750). Figure 1 shows the weekday and weekend activity counts of the six PA patterns (previously published [21]).

### PA correlates

PA correlates were selected *a priori* based on expert knowledge, availability on the CHMS survey, and scientific literature [22, 23]. Factors examined included: age (years), sex (2 levels: male, female), highest level of participant’s education (4 levels: less than high school, high school, some postsecondary, post-secondary), household income (3 levels: lowest/lower middle-income, upper middle-income, highest income), whether married and with children (4 levels: married and with child living at home, married without child living at home, not married and no child at home, not married and with child living at home), visible minority status and immigrant status (combined variable—4 levels: non-white immigrant, non-white (includes Indigenous people), non-immigrant, white immigrant, white non-immigrant), smoker status (2 levels: non-smoker, smoker), alcohol consumption (4 levels: regular, occasional, former drinker, never drank), weekly fruit and vegetable intake (servings, continuous), leisure time computer use (hours/week, continuous), leisure time watching TV/DVDs/videos (hours/week, continuous), leisure time playing video games (hours/week, continuous), employment status (2 levels: full-time, part-time), industry type (10 industries), self-perceived work stress (5 levels: not at all, not very, a bit, quite a bit, extremely), minimum skill level required for job (i.e., the nature of education and training required to be employed in the job) (5 levels: bachelor’s education required, college education or apprenticeship, secondary school, no training required), minimum physical strength required for job (2 levels: high – handling loads up to 20 kg, low – handling loads up to 10 kg or less), and work hours (hours/week, continuous).

### Analysis

Descriptive statistics (i.e., proportions and means) were used to examine variable distributions between those included and excluded for the analysis; Chi-square and t-tests were used to compare the samples.

The samples of the six PA pattern outcome categories were disproportionally distributed. Previous research has shown that this disproportionality results in high predictive accuracy for the majority class and poor predictive accuracy for the minority class and is best minimized by near-balanced distribution of groups [24]. As a result, we applied the Synthetic Minority Oversampling Technique (SMOTE) to adjust for sample size imbalances between the PA pattern outcome categories before the classification procedure [25]. The SMOTE method artificially generates new examples of the minority class using the nearest neighbors of these cases. The majority class examples are also under-sampled, leading to a more balanced dataset, which helps in improving the accuracy of classifiers of minority class groups and their performance in a decision tree model.

Classification and Regression Tree (CART) modelling [26] was used to classify and predict the association of various factors with workers’ daily PA patterns. CART-based decision trees were constructed by sequentially splitting the original sample based on factors of interest and then classifying individuals into distinct population subgroups that are relatively homogeneous with respect to a PA pattern outcome category. The split chosen for inclusion in the tree is the most error-reducing split across all potential factors. This recursive splitting process continues until the best split results are identified. Every factor is a potential candidate at each stage in growing the tree and some variables may appear several times using different cut-offs. A training set is used to learn a set of decision rules and a test set is then used to assess the performance of the grown decision tree. A CART tree was built from 80% of the original data (the training dataset) and then validated using the remaining 20% of the original sample (the test dataset). The tree was grown using the following hyperparameters: *minsplit*, the smallest number of observations in the parent node that could be split further, equal to 200; *minbucket*, the smallest number of observations that are allowed in a terminal node, equal to 200; and *complexity parameter* (cp; controls the size of the decision tree and selects the optimal tree size), equal to 0.00002. The tree was then pruned back to avoid overfitting the data by selecting a tree size that minimizes the cross validated error (the average error of the model on the test data). Variable importance scores were generated from the GINI (RMSE) measure, whereby variables with higher scores were considered most likely to be associated with the different PA patterns. The predictive performance of the final CART model was evaluated by the area under the ROC curve (AUC) as defined by multiple class classification problems [27]. The CART statistical procedures were performed using R version 4.2 and the ‘*Rpart’* package [28].

A multinomial logistic regression model (6 level outcome: lowest activity, moderate activity, fluctuating moderate activity, high daytime activity, moderate evening activity, highest activity) with stepwise variable selection was run and compared to the CART model output was run in both the training and test datasets to assess the relative performance of the two methods. The stepwise regression procedure was chosen over other regression procedures as its process of automatic searching of a subset of possible predictors of the outcome variable is comparable to the data-driven selection process of CART modelling and allows for the contribution of each variable to the model to be examined. The stepwise selection process consisted of a series of alternating forward selection and backward elimination steps with an unweighted training dataset, the former adds the same factors from the CART model selection to the model, while the latter removes variables from the model until a final fitted model is selected. At each step, variables were chosen based on the value of the likelihood score test and associated *p*-value at the 0.05 threshold. C-statistics from the stepwise regression model are equivalent to the AUC and were examined to measure the predictive accuracy and goodness-of-fit of the regression model. This part of the analyses was conducted using SAS version 9.4 (SAS Inc., Cary, NC).

## Results

A total of 8,909 participants were classified into six daily PA patterns. The characteristics of the study participants compared to the excluded sample (who had < 4 days of valid accelerometer data) is provided in Table 1 and the characteristics of participant groups within each of the six activity pattern categories are provided in Table 2; these characteristics are identical to those reported in a previous study [21]. Briefly, the analytical and excluded samples were similar except for age, marital status, and educational attainment. The analytical sample was, on average, slightly older (42 years vs. 39 years), had a greater proportion of married individuals (66% vs. 58%), and individuals with a postsecondary education (69% vs. 60%).

**Table 1:** Characteristics of participants included and excluded in the final analytical sample (survey weights applied)

	**Analytical sample**	**Excluded sample** ^a^	**Chi-square test on non-missing values**
	***N* = 8,909**	***N* = 1,673**	***P* value**
Sociodemographic variables			
Sex, %			0.05
Women	46.8	58.1	
Men	53.2	41.9	
Age, mean (SD)	42.4 (0.3)	38.5 (0.6)	< 0.001
Marital status and child living at home, %			0.004
Married and with child living at home	35.8	32.6	
Married without child living at home	29.8	25.2	
Not married and no child at home	30.1	38.7	
Not married and with child living at home	4.3	3.4	
Educational attainment, %			< 0.0001
Less than high school education	7.2	10.7	
High school diploma	21.6	22.1	
Some post-secondary education	2.7	7.3	
Post-secondary education	68.5	59.9	
Household income, %			0.08
Lowest/lower middle-income group	12.2	14.7	
Upper middle-income group	25.1	27.8	
Highest income group	62.7	57.5	
Minority and immigrant status, %			0.11
Non-white immigrant	16.4	18.5	
Non-white non-immigrant	6.2	8.6	
White immigrant	8.1	6.4	
White non-immigrant	69.2	66.6	
Province of residence			0.10
Alberta	14.1	12.1	
British Columbia	12.9	14.6	
Manitoba	0.9	0.9	
Newfoundland And Labrador	0.6	0.7	
New Brunswick	3.0	4.5	
Nova Scotia	1.8	1.3	
Ontario	40.3	38.6	
Prince Edward Island	0.6	0.7	
Quebec	21.1	24.5	
Saskatchewan	4.5	2.0	
Work variables			
Employment status, %			0.41
Full-time	82.1	80.5	
Part-time	17.9	19.5	
Hours worked per week, mean (SD)	39.1 (0.28)	39.7 (0.7)	0.46
Self-perceived work stress, %			0.21
Not at all	7.9	9.1	
Not very	19.5	22.0	
A bit	41.5	36.7	
Quite a bit	25.5	24.8	
Extremely	5.6	7.4	
Minimum physical strength required for job, %			0.21
High (requires handling of loads up to 20 kg)	26.6	30.2	
Low (requires handling of loads up to 10 kg or less)	73.4	69.8	
Minimum skill level required for job, %			0.04
Bachelor’s education required	23.7	19.0	
College education or apprenticeship	36.6	35.6	
Secondary school	26.7	31.7	
No training required	13.0	13.8	
Industry, %			0.002
Accommodation and food services	8.4	11.7	
Construction	6.8	9.2	
Education	7.5	4.8	
Health care & social assistance	11.9	8.6	
Manufacture	9.7	10.5	
Other goods industries	4.5	2.9	
Other service industries	26.8	27.7	
Public administration	4.8	5.6	
Retail Trade	11.9	11.0	
Trade & Transport	7.8	8.1	
Screen time, mean (SD)			
Hours per week on a computer	7.8 (0.2)	9.0 (0.5)	0.03
Hours per week playing video games	1.0 (0.1)	1.4 (0.2)	0.08
Hours per week watching television/DVDs/videos	10.2 (0.2)	10.2 (0.3)	1.00
Health behaviour variables			0.01
Fruit and vegetable intake per week, mean (SD)	26.6	30.2	
Smoking status, %			0.02
Non-smoker	80.0	75.0	
Smoker	20.0	25.0	
Alcohol consumption, %			0.51
Regular drinker	73.0	75.6	
Occasional drinker	13.7	12.8	
Former drinker	8.2	6.5	
Never drank	5.1	5.1	

*SD* Standard deviation

^a^Excluded sample based on incomplete accelerometer data (working sample with < 4 days of valid accelerometer data)

**Table 2:** Characteristics of daily physical activity patterns (survey weights applied)

	**Pattern 1**	**Pattern 2**	**Pattern 3**	**Pattern 4**	**Pattern 5**	**Pattern 6**
	***Lowest activity***	***Moderate consistent activity***	***Fluctuating moderate activity***	***High daytime activity***	***Moderate evening activity***	***Highest activity***
N	3,219	2,808	1,194	713	225	750
Mean activity counts per minute during a weekday (SD)	114.0 (1.2)	53.4 (0.9)	126.3 (1.8)	221.8 (5.3)	196.2 (7.3)	212.1 (5.9)
Daytime hours (SD)	183.2 (1.0)	84.9 (0.7)	198.8 (2.8)	357.2 (5.2)	232.5 (6.7)	318.9 (8.6)
Nighttime hours (SD)	44.7 (0.7)	21.9 (0.5)	53.8 (1.9)	86.4 (3.5)	160.0 (6.9)	105.4 (5.9)
Mean activity counts per minute during a weekend day (SD)	97.6 (1.6)	61.0 (1.8)	119.8 (8.5)	150.8 (6.0)	148.7 (10.1)	161.8 (6.4)
Daytime hours (SD)	160.8 (1.5)	99.2 (1.4)	192.9 (7.6)	245.7 (5.8)	190.5 (8.9)	271.4 (9.6)
Nighttime hours (SD)	34.4 (0.7)	22.8 (0.8)	46.7 (7.2)	55.9 (3.6)	107.0 (6.6)	52.3 (3.1)
Physically demanding job, %						
High (requires handling of loads up to 20 kg)	30.8	16.8	11.9	26.6	28.7	13.5
Low (requires handling of loads up to 10 kg)	69.2	83.2	88.1	73.4	71.3	86.5
Stationary job, %						
No (dynamic activities e.g., frequent walking)	67.7	52.0	45.2	57.6	81.8	47.0
Physically demanding job, %						
High (requires handling of loads up to 20 kg)	30.8	16.8	11.9	26.6	28.7	13.5
Self-reported physical activity						
Total minutes of recreational physical activity per week, mean (SD)	81.1 (7.5)	87.0 (8.0)	152.3 (17.7)	124.3 (15.9)	88.2 (33.1)	179.7 (17.7)
Proportion reporting recreational physical activity per week, %	52%	54%	79%	69%	44%	84%
Total minutes of other physical activity per week, mean, (SD)	278.6 (32.1)	156.4 (22.7)	99.9 (19.2)	190.7 (55.5)	443.3 (188.0)	103.5 (25.1)
Proportion reporting other physical activity per week, %	76%	66%	71%	64%	56%	71%

The SMOTE oversampling process improved the imbalance of the six PA groups used in the training dataset by increasing the size of the “fluctuating moderate activity” pattern by 1,131, the “high daytime activity” pattern by 1,344, the “moderate evening activity” pattern by 856, and the “highest activity” pattern by 1,438. Combining the original sample and the oversample (*N* = 13,678), the final analytical sample sizes were as follows: “lowest activity”: *N* = 2,808, “moderate consistent activity”: *N* = 3,219, “fluctuating moderate activity”: *N* = 2,325, “high daytime activity”: *N* = 2,057, “moderate evening activity”: *N* = 1,081, and “highest activity”: *N* = 2,188.

### Comparison of CART and regression techniques

Table 3 shows the confusion matrix output of the overall CART model performance in correctly and incorrectly classifying daily PA patterns. True positives from the model ranged from 0.26 to 0.32 and true negatives ranged from 0.03 to 0.24. The model’s micro-averaged F1 score (combined precision and recall accuracy) was 0.32.

**Table 3:** Confusion matrix of the classification accuracy of the CART model for multinomial physical activity patterns

	***Predicted physical activity pattern***					
**Actual physical activity pattern**	*Lowest activity*	*Moderate activity*	*Fluctuating moderate activity*	*High daytime activity*	*Moderate evening activity*	*Highest activity*
*Lowest activity*	0.23	0.31	0.19	0.16	0.11	0.16
*Moderate activity*	0.32	0.19	0.13	0.14	0.11	0.15
*Fluctuating moderate activity*	0.19	0.12	0.26	0.24	0.13	0.21
*High daytime activity*	0.13	0.15	0.18	0.26	0.20	0.14
*Moderate evening activity*	0.04	0.10	0.09	0.13	0.30	0.03
*Highest activity*	0.10	0.12	0.15	0.08	0.14	0.31

The CART tree performed marginally better than the stepwise multinomial logistic regression model, with an AUC value of the model predictability of the decision tree being 0.64 compared to the comparable AUC c-statistic of 0.63 for the multinomial logistic regression model. During the validation process, where the CART and regression models were applied to 80% of the training data and tested on 20% of the remaining data, the AUC of the CART model was 0.64 compared to the stepwise multinomial logistic regression model’s c-statistic of 0.55.

The factors selected in the final stepwise multinomial logistic regression model are provided in Supplementary Table 1. In addition to the same variables in the CART model, the stepwise model also included weekly leisure time watching TV/DVD/videos, weekly leisure time playing video games, full-time work status, visible minority and immigrant status as factors. The multinomial model included 156 estimates in total with 118 interaction terms between variables.

### Results of the CART tree

**Fig. 2:**
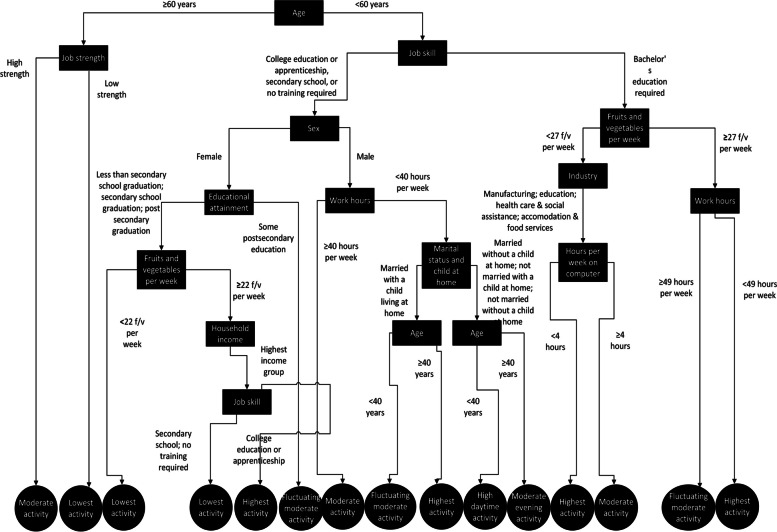
Classification and regression tree highlighting hierarchy of interrelated factors associated with daily physical activity patterns

Figure 2 shows the final CART tree highlighting the hierarchy of factors associated with each PA pattern category. The variables that were hierarchically most important to discriminate PA patterns were a participant’s age, the minimum physical strength required for the job and minimum skill level required for a person’s job. Participants aged 60 or above were associated with the “lowest activity” pattern if their job involved low physical strength. Participants aged 60 or above were associated with a “moderate activity” pattern if their job required high physical strength. All other factors were associated with workers aged less than 60 years and in order of hierarchy, are described as follows.

#### Lowest activity pattern

The “lowest activity” PA pattern was associated with three subgroups of people: i) those in a job requiring at least a college or secondary school education or no formal training, female, had a varied education level, and reported consuming less than 22 fruits/vegetables per week; ii) these people were similar to group; (i) except were in a job requiring only a secondary school or no formal training and were also in the highest household income group; (iii) consumed less than 22 fruits/ vegetables per week, were employed in the manufacturing, education, health care and social assistance, or accommodation and food services industries, and reported more than 4 h per week of recreational computer use.

#### Moderate activity pattern

The “moderate activity” pattern was associated with two subgroups: i) in a job requiring a college education/apprenticeship, high school education or no formal training, were male, and worked at least 40 h per week; ii) consumed less than 27 fruits/vegetables per week, were employed in the manufacturing, education, health care and social assistance, or accommodation and food services industries, and reported more than 4 h per week of leisure computer use.

#### Fluctuating moderate activity pattern

The “fluctuating moderate activity” pattern was associated with three subgroups: i) in a job requiring a college education/apprenticeship, high school education or no formal training, female, and had some postsecondary education; ii) in a job requiring a college education/apprenticeship, high school education or no formal training, were male, worked more than 40 h per week, were married and without a child living at home, and less than 40 years old; iii) in a job requiring at least a bachelor’s degree, consumed more than 27 fruits/vegetables per week, and worked 49 or more hours per week.

#### High daytime activity pattern

The “high daytime activity” pattern was associated with one subgroup: in a job requiring a college education/apprenticeship, high school education or no formal training, male, worked less than 40 h per week, were either married without a child living at home, unmarried with or without a child living at home, and was at least 46 years old.

#### Moderate evening activity pattern

The “moderate evening activity” pattern was associated with one subgroup, which was similar to the subgroup with a “high daytime activity” pattern, except they were under 46 years of age.

#### Highest activity pattern

The “highest activity” pattern was associated with three subgroups: i) female, had a varied education level, consumed 22 or more fruits/vegetables per week, and in a job requiring a college education or apprenticeship; ii) in a job requiring a college education/apprenticeship, high school education or no formal training, male, working less than 40 h per week, married and without a child living at home, and over 40 years of age; iii) in a job requiring at least a bachelor’s degree, consumed 27 or more fruits and vegetables per week, and worked less than 49 h a week.

## Discussion

This study applied a decision tree approach to understand the interrelated factors associated with working adults’ daily PA patterns and compared the model performance and factor selection to a conventional stepwise multinomial logistic regression approach. Participant’s age (whether older than 60 years of age or younger), the minimum physical strength and minimum skill level required for a person’s job were found to be the most important discriminating factors of daily PA patterns. The decision tree highlighted three distinct homogenous subgroups associated with the “lowest activity pattern”, three subgroups associated with a “moderate activity” pattern, three subgroups associated with the “fluctuating moderate activity” pattern, one subgroup for the “high daytime” and “moderate evening activity” patterns, and four subgroups for the “highest activity” pattern. The factors associated with workers’ PA patterns from the decision tree model included the minimum job skill and job strength levels, sex, educational attainment, fruit/vegetable intake, industry type, work hours, marital status and having a child living at home, computer screen time, and household income group. The CART tree performed similarly to the stepwise logistic regression model with the same input variables. However, the CART approach provided a more parsimonious model with fewer factors than the stepwise approach and showed the hierarchical importance of the selected factors. Accordingly, the CART approach may be a more practical approach compared to convention regression to inform interventions that recognise the complex interrelated factors associated with daily PA in workers, particularly with outcome variables that have three or more categories.

Other studies that have used a decision tree approach identified occupation-type as an important factor related to PA participation. Lakerveld et al. found current occupation type to be the most prominent discriminator between adults being physically active and inactive [16], while Farrahi et al. found that workers in some physically demanding occupations (i.e., forestry workers, farmers, and process and transport workers) were at the least risk of being physically inactive [14]. The present study’s exploration of work-related factors provides a more nuanced explanation of the contribution of occupations to PA behaviours and found that some work-related factors were prominent in all subgroups associated with daily PA patterns. However, job skill level (nature of education and training required to be employed in an occupation) was among the higher order factors associated with PA, supporting studies showing that workers with higher skill discretion (breadth of skills usable on the job) and decision latitude (a worker’s potential control over their tasks) were associated with higher leisure time PA [29, 30]. Work hours also featured prominently in the different subgroups, with workers with shorter work hours associated with patterns of higher daily PA. Workers in subgroups with long work hours were associated with moderate patterns of daily PA but were not associated with the “lowest activity” pattern. As these groups undertook their PA mostly during daytime hours, it is possible that these workers accumulated most of their PA at work and accumulated little PA outside work perhaps due to a perceived lack of time due to the responsibilities and demands of work [31].

Age was the highest-order factor in the CART model to differentiate daily PA patterns and two of the three occurrences of the “lowest activity” pattern occurred among workers ≥ 60 years. Studies have demonstrated that PA levels decline over the life course, whereby levels are stable or increase in younger adulthood and begin to decline around middle age (40 to 45 years) and up to retirement [8–10]. Declining PA levels due to advancing age can be attributed to a lack of time for PA because of increasing work and family responsibilities, and the onset of health and functional problems [8, 11]. As the “lowest activity” pattern also occurred among older workers in low physical strength jobs suggests that promoting increasing PA levels in and outside the workplace can be an important PA intervention target for older workers. Among workers < 60 years, a subgroup of females in the “lowest” and “highest activity” pattern groups were differentiated only by their job skill level, whereby the “highest activity” group had higher job skill levels. It is possible that workers with higher job skill levels have more decision-making authority in their jobs, which is associated with higher motivation for leisure time PA attainment [32].

While the CART tree model performed marginally better to the stepwise multinomial logistic regression model, their overall classification capabilities were poor and moderate based on their AUC and C-statistic, respectively. The model performance could be improved by using a larger dataset and with a greater selection of potential factors related to PA. However, the CART model provided a more parsimonious model with fewer factors selected. In comparison, the stepwise regression approach was a highly complex model with 100 s of parameters and interactions and interpreting the model would be difficult to interpret compared to the CART approach, especially for three or more outcome categories. With the CART model also providing additional detail on the hierarchical importance of each factor and the interrelationships between factors, the study findings and future decision tree approaches on daily PA patterns may inform precision public health approaches to identify optimal interventions for specific and homogenous sub-populations. For example, targeting low job strength/sedentary occupations, a higher order factor, may be a priority when targeting older workers, while interventions targeted towards specific occupations, or for men and women may be more effective for promoting daily PA among younger worker populations.

### Strengths and limitations

This study has several strengths. The study uses an easily interpretable decision tree approach that supports the straightforward interpretation of the complex interactions of PA correlates. The use of the CHMS also provides a population-based sample in which continuous device-measured PA was collected and provides more accurate reflections of daily PA patterns compared to the more subjective self-reported PA data [33]. The use of the SMOTE oversampling technique to estimate the interrelated factors associated with six different daily PA patterns of workers that were previously identified through a hierarchical cluster analysis machine-learning approach is also a strength as most previous studies have focused on whether participants adhered to PA recommendations. The advantage of this approach is that it alleviates overfitting caused by random sampling as synthetic examples are generated without any loss of information [34]. However, a limitation of the SMOTE approach is that it is difficult to determine the number of nearest neighbors, and there is strong blindness in the selection of nearest neighbors for the synthetic examples [35].

PA of any type and duration is widely recognised to be beneficial to health, however, there is evidence of a possible PA health paradox whereby the health effects of PA are different depending on the content in which it is accumulated and in particular, high levels of occupational PA has been linked to health risks [36]. While the focus of this study was to examine the factors associated with different daily PA patterns, we had previously demonstrated the possibility of a PA health paradox among workers with a “high daytime activity pattern” (PA was likely undertaken during work hours) as these individuals were the only group to not have a reduced risk of 10-year CVD compared to the “lowest activity pattern” [21]. Accordingly, it is possible that workers with a “high daytime activity pattern” may not have CVD health benefits from interventions promoting increased PA. The analysis of potential correlates was limited by their availability, for example, information on participants’ time spent outdoors, the quality of the neighbourhood work environment, whether participants worked from home, and behavioural correlates were either not collected in the CHMS or not measured in all survey cycles. While other studies have included current occupation as a potential correlate in decision tree models, our use of the national occupation classification taxonomy to inform ratings of typical physical strengths and job skills required for an occupation provides more detail on the work-related factors (work-related fatigue and job demands-control) that are plausibly related to PA attainment. The CHMS is also a cross-sectional dataset and causality cannot be determined. Furthermore, the CART and stepwise regression approaches include all factors in a single model without explicitly accounting for some factors being causally antecedent or on each others’ causal pathways. The interpretation of continuous variables in the CART methodology is also a limitation, whereby a “split” in a tree is based on arbitrary mathematical conditions which may not reflect widely applied thresholds or recommendations such as the varied cut-offs for fruit/vegetable intake, screen time, and weekly work hours. We contemplated generating random forests, which is a collection of decision trees with each tree fitted from a bootstrap sample of the original data as this may have generated a more accurate prediction model. However, random forests lose the interpretability of individual decision trees [37].

In conclusion, using a CART decision tree modelling approach, we identified subgroups of interacting factors associated with six daily patterns of PA in Canadian workers. Work-related factors, particularly age, job skill level, and physical strength used on the job appeared as the most important discriminators of daily PA patterns, particularly related to differences related to sex, work hours, and industry type. While further improvements of the CART tree model are needed, the approach warrants consideration as a method for identifying the interrelated factors associated with daily PA patterns.

### Supplementary Information

**Additional file 1: Supplementary Table 1. **Output from final stepwise multinomial logistic regression model used to identify factors associated with the outcome of six physical activity pattern categories.

**Additional file 2.** STROBE Statement—checklist of items that should be included in reports of observational studies.

## Data Availability

The datasets generated and/or analysed during the current study are available in the Canadian Health Measures Survey (CHMS) repository, https://www23.statcan.gc.ca/imdb/p2SV.pl?Function=getSurvey&Id=10263.

## Abbreviations

CART: Classification and Regression Tree
PA: Physical Activity
CHMS: Canadian Health Measures Survey
SMOTE: Synthetic Minority Oversampling Technique
