# Supplementary material for: Identifying the sociodemographic and work-related factors related to workers’ daily physical activity using a decision tree approach
Source: BMC Public Health. 2023 Sep 23;23:1853. doi: 10.1186/s12889-023-16747-9 (PMC10517528; doi:10.1186/s12889-023-16747-9)
Supplement: Supplementary file 1 — Additional file 1: Supplementary Table 1. Output from final stepwise multinomial logistic regression model used to identify factors associated with the outcome of six physical activity pattern categories. [file 12889_2023_16747_MOESM1_ESM.docx]

**Supplementary Table 1.** Output from final stepwise multinomial logistic regression model used to identify factors associated with the outcome of six physical activity pattern categories.

| **Exposure variable** | **Variable categories** | **Variable categories** | **Beta Estimate** | **Standard** | **Wald** | **Pr > Chi Sq** |
| --- | --- | --- | --- | --- | --- | --- |
|  |  |  |  | **Error** | **Chi-Square** |  |
| Intercept | Highest activity pattern |  | 0.81 | 1.21 | 0.45 | 0.50 |
| Intercept | Moderate evening activity pattern |  | 1.11 | 1.21 | 0.85 | 0.36 |
| Intercept | High daytime activity pattern |  | 1.80 | 1.21 | 2.22 | 0.14 |
| Intercept | Fluctuating moderate activity pattern |  | 2.58 | 1.21 | 4.54 | 0.03 |
| Intercept | Moderate activity pattern |  | 4.25 | 1.21 | 12.30 | 0.00 |
| Age |  |  | -0.04 | 0.01 | 13.78 | 0.00 |
| Sex |  |  | -1.05 | 0.25 | 17.47 | <.0001 |
| Whether married and with children | Married w/ child |  | -0.29 | 0.33 | 0.77 | 0.38 |
| Whether married and with children | Married w/o child |  | -0.18 | 0.41 | 0.20 | 0.65 |
| Whether married and with children | Not Married w/ child |  | -0.84 | 0.51 | 2.74 | 0.10 |
| Educational attainment |  |  | 0.00 | 0.02 | 0.01 | 0.94 |
| Household income |  |  | 0.12 | 0.30 | 0.17 | 0.68 |
| Visible minority and immigrant status | Non-white immigrant |  | -0.49 | 0.42 | 1.31 | 0.25 |
| Visible minority and immigrant status | Non-white non-immigrant |  | -0.54 | 0.63 | 0.73 | 0.39 |
| Visible minority and immigrant status | White immigrant |  | 1.14 | 0.68 | 2.81 | 0.09 |
| Smoker |  |  | 0.73 | 0.54 | 1.82 | 0.18 |
| Alcohol consumption | Former drinker |  | -0.50 | 0.67 | 0.57 | 0.45 |
| Alcohol consumption | Occasional drinker |  | -0.30 | 0.61 | 0.25 | 0.62 |
| Alcohol consumption | Regular drinker |  | -0.33 | 0.56 | 0.35 | 0.55 |
| Weekly fruit/veg intake |  |  | 0.01 | 0.01 | 1.06 | 0.30 |
| Weekly leisure time computer use |  |  | -0.01 | 0.01 | 1.72 | 0.19 |
| Weekly leisure time watching TV/DVDs/videos |  |  | -0.02 | 0.01 | 13.47 | 0.00 |
| Weekly leisure time playing video games |  |  | 0.00 | 0.02 | 0.08 | 0.77 |
| Full time work |  |  | -0.33 | 0.39 | 0.70 | 0.40 |
| Industry type | Accommodation and food services |  | 0.24 | 0.62 | 0.15 | 0.70 |
| Industry type | Construction |  | 0.14 | 0.76 | 0.03 | 0.86 |
| Industry type | Education |  | -0.87 | 0.75 | 1.36 | 0.24 |
| Industry type | Health care & social assistance |  | 0.05 | 0.64 | 0.01 | 0.94 |
| Industry type | Manufacture |  | 0.21 | 0.70 | 0.09 | 0.76 |
| Industry type | Other goods industries |  | -0.60 | 0.96 | 0.40 | 0.53 |
| Industry type | Other service industries |  | -0.40 | 0.52 | 0.60 | 0.44 |
| Industry type | Public administration |  | -0.17 | 1.00 | 0.03 | 0.86 |
| Industry type | Trade & Transport |  | -0.77 | 0.71 | 1.19 | 0.27 |
| Physical strength req for job | High |  | 0.46 | 0.29 | 2.60 | 0.11 |
| Minimum skill req for job | Bachelor’s education required |  | -1.15 | 0.50 | 5.34 | 0.02 |
| Minimum skill req for job | College education or apprenticeship |  | -0.95 | 0.41 | 5.24 | 0.02 |
| Minimum skill req for job | Secondary school |  | -0.33 | 0.42 | 0.63 | 0.43 |
| Work hours |  |  | -0.01 | 0.01 | 0.21 | 0.65 |
| Sex*Industry type | Accommodation and food services |  | -0.09 | 0.20 | 0.22 | 0.64 |
| Sex*Industry type | Construction |  | -0.25 | 0.30 | 0.69 | 0.41 |
| Sex*Industry type | Education |  | -0.07 | 0.21 | 0.12 | 0.73 |
| Sex*Industry type | Health care & social assistance |  | -0.14 | 0.20 | 0.48 | 0.49 |
| Sex*Industry type | Manufacturing |  | -0.08 | 0.21 | 0.16 | 0.69 |
| Sex*Industry type | Other goods industries |  | 0.43 | 0.28 | 2.47 | 0.12 |
| Sex*Industry type | Other service industries |  | -0.11 | 0.16 | 0.42 | 0.52 |
| Sex*Industry type | Public administration |  | -0.07 | 0.23 | 0.10 | 0.75 |
| Sex*Industry type | Trade & Transport |  | 0.23 | 0.22 | 1.05 | 0.30 |
| Household income*Industry type | Accommodation and food services |  | -0.03 | 0.12 | 0.05 | 0.83 |
| Household income*Industry type | Construction |  | 0.01 | 0.14 | 0.00 | 0.96 |
| Household income*Industry type | Education |  | 0.12 | 0.15 | 0.59 | 0.44 |
| Household income*Industry type | Health care & social assistance |  | -0.06 | 0.12 | 0.25 | 0.62 |
| Household income*Industry type | Manufacturing |  | 0.00 | 0.13 | 0.00 | 1.00 |
| Household income*Industry type | Other goods industries |  | 0.21 | 0.18 | 1.40 | 0.24 |
| Household income*Industry type | Other service industries |  | 0.03 | 0.10 | 0.11 | 0.75 |
| Household income*Industry type | Public administration |  | 0.20 | 0.21 | 0.99 | 0.32 |
| Household income*Industry type | Trade & Transport |  | 0.14 | 0.14 | 0.95 | 0.33 |
| Smoker*Industry type | Accommodation and food services |  | -0.42 | 0.23 | 3.34 | 0.07 |
| Smoker*Industry type | Construction |  | -0.30 | 0.24 | 1.54 | 0.21 |
| Smoker*Industry type | Education |  | -0.39 | 0.28 | 1.87 | 0.17 |
| Smoker*Industry type | Health care & social assistance |  | 0.04 | 0.23 | 0.03 | 0.85 |
| Smoker*Industry type | Manufacturing |  | -0.58 | 0.22 | 6.71 | 0.01 |
| Smoker*Industry type | Other goods industries |  | -0.41 | 0.30 | 1.90 | 0.17 |
| Smoker*Industry type | Other service industries |  | -0.28 | 0.19 | 2.15 | 0.14 |
| Smoker*Industry type | Public administration |  | 0.05 | 0.31 | 0.02 | 0.87 |
| Smoker*Industry type | Trade & Transport |  | -0.38 | 0.24 | 2.48 | 0.12 |
| Weekly fruit/veg intake*Industry type | Accommodation and food services |  | -0.01 | 0.01 | 0.58 | 0.45 |
| Weekly fruit/veg intake*Industry type | Construction |  | 0.00 | 0.01 | 0.02 | 0.88 |
| Weekly fruit/veg intake*Industry type | Education |  | -0.01 | 0.01 | 0.66 | 0.42 |
| Weekly fruit/veg intake*Industry type | Health care & social assistance |  | 0.01 | 0.01 | 1.05 | 0.31 |
| Weekly fruit/veg intake*Industry type | Manufacturing |  | -0.01 | 0.01 | 0.94 | 0.33 |
| Weekly fruit/veg intake*Industry type | Other goods industries |  | 0.00 | 0.01 | 0.27 | 0.60 |
| Weekly fruit/veg intake*Industry type | Other service industries |  | 0.01 | 0.01 | 1.46 | 0.23 |
| Weekly fruit/veg intake*Industry type | Public administration |  | 0.01 | 0.01 | 0.76 | 0.38 |
| Weekly fruit/veg intake*Industry type | Trade & Transport |  | -0.01 | 0.01 | 1.97 | 0.16 |
| Full time*Industry type | Accommodation and food services |  | 0.34 | 0.20 | 2.92 | 0.09 |
| Full time*Industry type | Construction |  | 0.08 | 0.30 | 0.06 | 0.80 |
| Full time*Industry type | Education |  | 0.87 | 0.22 | 15.45 | <.0001 |
| Full time*Industry type | Health care & social assistance |  | 0.30 | 0.19 | 2.38 | 0.12 |
| Full time*Industry type | Manufacturing |  | 0.25 | 0.33 | 0.55 | 0.46 |
| Full time*Industry type | Other goods industries |  | -0.63 | 0.44 | 2.02 | 0.16 |
| Full time*Industry type | Other service industries |  | 0.21 | 0.17 | 1.49 | 0.22 |
| Full time*Industry type | Public administration |  | -0.21 | 0.34 | 0.41 | 0.52 |
| Full time*Industry type | Trade & Transport |  | 0.09 | 0.27 | 0.10 | 0.75 |
| Physical strength req for job*Industry type | High | Accommodation and food services | -0.07 | 0.28 | 0.05 | 0.81 |
| Physical strength req for job*Industry type | High | Construction | 0.06 | 0.24 | 0.06 | 0.81 |
| Physical strength req for job*Industry type | High | Education | 0.28 | 0.35 | 0.67 | 0.41 |
| Physical strength req for job*Industry type | High | Health care & social assistance | -0.11 | 0.22 | 0.24 | 0.62 |
| Physical strength req for job*Industry type | High | Manufacture | 0.05 | 0.23 | 0.05 | 0.83 |
| Physical strength req for job*Industry type | High | Other goods industries | 0.13 | 0.28 | 0.22 | 0.64 |
| Physical strength req for job*Industry type | High | Other service industries | 0.05 | 0.21 | 0.06 | 0.80 |
| Physical strength req for job*Industry type | High | Public administration | -0.56 | 0.29 | 3.77 | 0.05 |
| Physical strength req for job*Industry type | High | Trade & Transport | 0.39 | 0.24 | 2.54 | 0.11 |
| Physical strength req for job*Visible minority and immigrant status | High | non-white immigrant | 0.17 | 0.14 | 1.38 | 0.24 |
| Physical strength req for job*Visible minority and immigrant status | High | non-white non-immigrant | -0.19 | 0.22 | 0.75 | 0.39 |
| Physical strength req for job*Visible minority and immigrant status | High | white immigrant | -0.04 | 0.19 | 0.05 | 0.82 |
| Weekly leisure time computer use*Physical strength req for job | High |  | 0.00 | 0.01 | 0.00 | 0.95 |
| Full time*Physical strength req for job | High |  | -0.08 | 0.14 | 0.29 | 0.59 |
| Physical strength req for job*Minimum skill req for job | High | Bachelor’s education required | 0.10 | 0.37 | 0.08 | 0.78 |
| Physical strength req for job*Minimum skill req for job | High | College education or apprenticeship | -0.33 | 0.16 | 4.35 | 0.04 |
| Physical strength req for job*Minimum skill req for job | High | Secondary school | -0.21 | 0.16 | 1.67 | 0.20 |
| Age*Weekly fruit/veg intake |  |  | 0.00 | 0.00 | 1.24 | 0.27 |
| Household income*Weekly fruit/veg intake |  |  | 0.00 | 0.00 | 4.07 | 0.04 |
| Weekly fruit/veg intake*Weekly leisure time computer use |  |  | 0.00 | 0.00 | 0.91 | 0.34 |
| Weekly fruit/veg intake*Weekly leisure time playing video games |  |  | 0.00 | 0.00 | 5.21 | 0.02 |
| Age*Work hours |  |  | 0.00 | 0.00 | 1.64 | 0.20 |
| Sex*Work hours |  |  | 0.01 | 0.00 | 9.96 | 0.00 |
| Household income*Work hours |  |  | -0.01 | 0.00 | 6.52 | 0.01 |
| Work hours*Visible minority and immigrant status | Non-white immigrant |  | -0.02 | 0.00 | 11.23 | 0.00 |
| Work hours*Visible minority and immigrant status | Non-white non-immigrant |  | -0.01 | 0.01 | 0.70 | 0.40 |
| Work hours*Visible minority and immigrant status | White immigrant |  | -0.02 | 0.01 | 9.30 | 0.00 |
| Work hours*Alcohol consumption | Former drinker |  | 0.01 | 0.01 | 0.58 | 0.45 |
| Work hours*Alcohol consumption | Occasional drinker |  | 0.00 | 0.01 | 0.43 | 0.51 |
| Work hours*Alcohol consumption | Regular drinker |  | 0.00 | 0.01 | 0.28 | 0.60 |
| Weekly leisure time playing video games*Work hours |  |  | 0.00 | 0.00 | 0.68 | 0.41 |
| Sex |  |  | 0.00 | 0.00 | 1.36 | 0.24 |
| Age*Whether married and with children | Married w/ child |  | 0.01 | 0.00 | 7.18 | 0.01 |
| Age*Whether married and with children | Married w/o child |  | -0.01 | 0.00 | 2.56 | 0.11 |
| Age*Whether married and with children | Not Married w/ child |  | 0.00 | 0.01 | 0.04 | 0.84 |
| Age*Household income |  |  | 0.00 | 0.00 | 1.60 | 0.21 |
| Age*Visible minority and immigrant status | Non-white immigrant |  | 0.01 | 0.01 | 3.83 | 0.05 |
| Age*Visible minority and immigrant status | Non-white non-immigrant |  | 0.00 | 0.01 | 0.47 | 0.49 |
| Age*Visible minority and immigrant status | White immigrant |  | -0.02 | 0.01 | 6.61 | 0.01 |
| Age*Weekly leisure time playing video games |  |  | 0.00 | 0.00 | 0.00 | 0.97 |
| Sex*Minimum skill req for job | Bachelor’s education required |  | 0.14 | 0.15 | 0.85 | 0.36 |
| Sex*Minimum skill req for job | College education or apprenticeship |  | 0.25 | 0.14 | 2.95 | 0.09 |
| Sex*Minimum skill req for job | Secondary school |  | 0.08 | 0.15 | 0.33 | 0.57 |
| Household income*Minimum skill req for job | Bachelor’s education required |  | 0.32 | 0.11 | 7.70 | 0.01 |
| Household income*Minimum skill req for job | College education or apprenticeship |  | 0.17 | 0.09 | 3.51 | 0.06 |
| Household income*Minimum skill req for job | Secondary school |  | 0.09 | 0.09 | 0.93 | 0.34 |
| Household income*Whether married and with children | Married w/ child |  | -0.05 | 0.07 | 0.46 | 0.50 |
| Household income*Whether married and with children | Married w/o child |  | 0.16 | 0.09 | 2.95 | 0.09 |
| Household income*Whether married and with children | Not Married w/ child |  | 0.18 | 0.12 | 2.10 | 0.15 |
| Weekly leisure time computer use*Whether married and with children | Married w/ child |  | 0.00 | 0.01 | 0.47 | 0.49 |
| Weekly leisure time computer use*Whether married and with children | Married w/o child |  | 0.00 | 0.01 | 0.41 | 0.52 |
| Weekly leisure time computer use*Whether married and with children | Not Married w/ child |  | 0.03 | 0.01 | 8.21 | 0.00 |
| Household income*Alcohol consumption | Former drinker |  | -0.05 | 0.16 | 0.10 | 0.76 |
| Household income*Alcohol consumption | Occasional drinker |  | -0.16 | 0.14 | 1.19 | 0.28 |
| Household income*Alcohol consumption | Regular drinker |  | -0.03 | 0.13 | 0.06 | 0.81 |
| Household income*Weekly leisure time playing video games |  |  | 0.00 | 0.00 | 0.26 | 0.61 |
| Household income*Full time |  |  | -0.09 | 0.11 | 0.70 | 0.40 |
| Sex*Smoker |  |  | -0.10 | 0.12 | 0.73 | 0.39 |
| Smoker*Alcohol consumption | Former drinker |  | -0.30 | 0.52 | 0.34 | 0.56 |
| Smoker*Alcohol consumption | Occasional drinker |  | -0.64 | 0.50 | 1.63 | 0.20 |
| Smoker*Alcohol consumption | Regular drinker |  | -0.69 | 0.48 | 2.06 | 0.15 |
| Smoker*Weekly leisure time playing video games |  |  | 0.02 | 0.01 | 6.02 | 0.01 |
| Sex*Visible minority and immigrant status | Non-white immigrant |  | -0.08 | 0.13 | 0.39 | 0.53 |
| Sex*Visible minority and immigrant status | Non-white non-immigrant |  | -0.44 | 0.18 | 5.74 | 0.02 |
| Sex*Visible minority and immigrant status | White immigrant |  | -0.21 | 0.16 | 1.73 | 0.19 |
| Visible minority and immigrant status*Alcohol consumption | Non-white immigrant | Former drinker | 0.60 | 0.30 | 3.90 | 0.05 |
| Visible minority and immigrant status*Alcohol consumption | Non-white immigrant | Occasional drinker | 1.13 | 0.28 | 16.56 | <.0001 |
| Visible minority and immigrant status*Alcohol consumption | Non-white immigrant | Regular drinker | 0.67 | 0.26 | 6.85 | 0.01 |
| Visible minority and immigrant status*Alcohol consumption | Non-white non-immigrant | Former drinker | 1.23 | 0.54 | 5.28 | 0.02 |
| Visible minority and immigrant status*Alcohol consumption | Non-white non-immigrant | Occasional drinker | 1.37 | 0.48 | 8.03 | 0.00 |
| Visible minority and immigrant status*Alcohol consumption | Non-white non-immigrant | Regular drinker | 1.22 | 0.45 | 7.25 | 0.01 |
| Visible minority and immigrant status*Alcohol consumption | White immigrant | Former drinker | 0.78 | 0.61 | 1.65 | 0.20 |
| Visible minority and immigrant status*Alcohol consumption | White immigrant | Occasional drinker | 1.01 | 0.58 | 3.05 | 0.08 |
| Visible minority and immigrant status*Alcohol consumption | White immigrant | Regular drinker | 0.63 | 0.55 | 1.34 | 0.25 |
