# Supplementary material for: Identifying the sociodemographic and work-related factors related to workers’ daily physical activity using a decision tree approach
Source: BMC Public Health. 2023 Sep 23;23:1853. doi: 10.1186/s12889-023-16747-9 (PMC10517528; doi:10.1186/s12889-023-16747-9)
Supplement: Supplementary file 2 — Additional file 2. STROBE Statement—checklist of items that should be included in reports of observational studies. [file 12889_2023_16747_MOESM2_ESM.docx]

STROBE Statement—checklist of items that should be included in reports of observational studies

|  | Item No. | Recommendation | Page  No. | Relevant text from manuscript |
| --- | --- | --- | --- | --- |
| **Title and abstract** | 1 | (*a*) Indicate the study’s design with a commonly used term in the title or the abstract | 1 | “Identifying the sociodemographic and work-related factors related to workers’ daily physical activity *using a decision tree approach*” |
|  |  | (*b*) Provide in the abstract an informative and balanced summary of what was done and what was found | 2 | “*Classification and Regression Tree (CART) modelling was used to identify the discriminators associated with six daily physical activity patterns*. The performance of the CART approach was compared to a stepwise multinomial logistic regression model.  Results: *Among the 8,909 workers analysed, the most important CART discriminators of daily physical activity patterns were age, job skill, and physical strength requirements of the job.”* |
| Introduction | | | |  |
| Background/rationale | 2 | Explain the scientific background and rationale for the investigation being reported | 3 | Background section |
| Objectives | 3 | State specific objectives, including any prespecified hypotheses | 6 | *“This study used a decision tree machine learning approach to examine the hierarchy of interrelated factors associated with daily PA behaviour patterns in a population-based sample of Canadian workers. The performance of the decision tree approach was compared to a conventional regression approach.”* |
| Methods | | | |  |
| Study design | 4 | Present key elements of study design early in the paper | 6 | “The study examined cross-sectional data from the Canadian Health Measures Survey (CHMS), which has a sampling frame representative of >96% of the Canadian population. Data was collected every two years from a sample of approximately 5,000 Canadians aged 6 to 79 years (20). The CHMS includes self-reported demographics and health data, accelerometer-measured continuous PA data and clinically measured cardiometabolic markers. Details on the survey design and data collection methods have been described previously (20).” |
| Setting | 5 | Describe the setting, locations, and relevant dates, including periods of recruitment, exposure, follow-up, and data collection | 6-7 | “The study examined cross-sectional data from the Canadian Health Measures Survey (CHMS), which has a sampling frame representative of >96% of the Canadian population. Data was collected every two years from a sample of approximately 5,000 Canadians aged 6 to 79 years (20). The CHMS includes self-reported demographics and health data, accelerometer-measured continuous PA data and clinically measured cardiometabolic markers. Details on the survey design and data collection methods have been described previously (20).  Five cycles of cross-sectional CHMS data (2007 to 2017) from ~11,250 respondents (49% female) were combined. Participants were included if they were 18 years of age or older, reported having a job, and were at work in the week prior to responding to the survey. Pregnant women were excluded from the sample. A total of 10,582 participants met the inclusion criteria. Among them, 8,909 participants had valid accelerometer data; activity data for 10 hours per day and for at least 4 days (participants were told to wear accelerometers on their waist for a week except when sleeping, swimming, or bathing).” |
| Participants | 6 | (*a*) *Cohort study*—Give the eligibility criteria, and the sources and methods of selection of participants. Describe methods of follow-up  *Case-control study*—Give the eligibility criteria, and the sources and methods of case ascertainment and control selection. Give the rationale for the choice of cases and controls  *Cross-sectional study*—Give the eligibility criteria, and the sources and methods of selection of participants | 6-7 | Five cycles of cross-sectional CHMS data (2007 to 2017) from ~11,250 respondents (49% female) were combined. Participants were included if they were 18 years of age or older, reported having a job, and were at work in the week prior to responding to the survey. Pregnant women were excluded from the sample. A total of 10,582 participants met the inclusion criteria. Among them, 8,909 participants had valid accelerometer data; activity data for 10 hours per day and for at least 4 days (participants were told to wear accelerometers on their waist for a week except when sleeping, swimming, or bathing). |
|  |  | (*b*) *Cohort study*—For matched studies, give matching criteria and number of exposed and unexposed  *Case-control study*—For matched studies, give matching criteria and the number of controls per case |  |  |
| Variables | 7 | Clearly define all outcomes, exposures, predictors, potential confounders, and effect modifiers. Give diagnostic criteria, if applicable | 7-8 | Subsections on “Outcome: Daily physical activity (PA) patterns”, and “PA correlates”. |
| Data sources/ measurement | 8* | For each variable of interest, give sources of data and details of methods of assessment (measurement). Describe comparability of assessment methods if there is more than one group | *7-8* | Subsections on “Outcome: Daily physical activity (PA) patterns”, and “PA correlates”. |
| Bias | 9 | Describe any efforts to address potential sources of bias | 9 | “The samples of the six PA pattern outcome categories were disproportionally distributed. Previous research has shown that this disproportionality results in high predictive accuracy for the majority class and poor predictive accuracy for the minority class and is best minimized by near-balanced distribution of groups (24). As a result, we applied the Synthetic Minority Oversampling Technique (SMOTE) to adjust for sample size imbalances between the PA pattern outcome categories before the classification procedure (25). *The SMOTE method artificially generates new examples of the minority class using the nearest neighbors of these cases. The majority class examples are also under-sampled, leading to a more balanced dataset, which helps in improving the accuracy of classifiers of minority class groups and their performance in a decision tree model.”* |
| Study size | 10 | Explain how the study size was arrived at | 12 | “The SMOTE oversampling process improved the imbalance of the six PA groups used in the training dataset by increasing the size of the “fluctuating moderate activity” pattern by 1,131, the “high daytime activity” pattern by 1,344, the “moderate evening activity” pattern by 856, and the “highest activity” pattern by 1,438. Combining the original sample and the oversample (N=13,678), the final analytical sample sizes were as follows: “lowest activity”: N = 2,808, “moderate consistent activity”: N = 3,219, “fluctuating moderate activity”: N = 2,325, “high daytime activity”: N = 2,057, “moderate evening activity”: N = 1,081, and “highest activity”: N = 2,188.” |

Continued on next page

| Quantitative variables | 11 | Explain how quantitative variables were handled in the analyses. If applicable, describe which groupings were chosen and why | 9 | Analysis section |
| --- | --- | --- | --- | --- |
| Statistical methods | 12 | (*a*) Describe all statistical methods, including those used to control for confounding | 9-10 | Analysis section |
|  |  | (*b*) Describe any methods used to examine subgroups and interactions | 9-10 | Analysis section |
|  |  | (*c*) Explain how missing data were addressed | 9-10 | Analysis section |
|  |  | (*d*) *Cohort study*—If applicable, explain how loss to follow-up was addressed  *Case-control study*—If applicable, explain how matching of cases and controls was addressed  *Cross-sectional study*—If applicable, describe analytical methods taking account of sampling strategy |  |  |
|  |  | (*e*) Describe any sensitivity analyses |  |  |
| Results | | | | |
| Participants | 13* | (a) Report numbers of individuals at each stage of study—eg numbers potentially eligible, examined for eligibility, confirmed eligible, included in the study, completing follow-up, and analysed | 11-12 | “A total of 8,909 participants were classified into six daily PA patterns. The characteristics of the study participants compared to the excluded sample (who had <4 days of valid accelerometer data) is provided in Table 1 and the characteristics of participant groups within each of the six activity pattern categories are provided in Table 2; these characteristics are identical to those reported in a previous study (21). Briefly, the analytical and excluded samples were similar except for age, marital status, and educational attainment. The analytical sample was, on average, slightly older (42 years vs. 39 years), had a greater proportion of married individuals (66% vs. 58%), and individuals with a postsecondary education (69% vs. 60%).  The SMOTE oversampling process improved the imbalance of the six PA groups used in the training dataset by increasing the size of the “fluctuating moderate activity” pattern by 1,131, the “high daytime activity” pattern by 1,344, the “moderate evening activity” pattern by 856, and the “highest activity” pattern by 1,438. Combining the original sample and the oversample (N=13,678), the final analytical sample sizes were as follows: “lowest activity”: N = 2,808, “moderate consistent activity”: N = 3,219, “fluctuating moderate activity”: N = 2,325, “high daytime activity”: N = 2,057, “moderate evening activity”: N = 1,081, and “highest activity”: N = 2,188.” |
|  |  | (b) Give reasons for non-participation at each stage |  | N/A |
|  |  | (c) Consider use of a flow diagram |  |  |
| Descriptive data | 14* | (a) Give characteristics of study participants (eg demographic, clinical, social) and information on exposures and potential confounders | 11-12 | “A total of 8,909 participants were classified into six daily PA patterns. The characteristics of the study participants compared to the excluded sample (who had <4 days of valid accelerometer data) is provided in Table 1 and the characteristics of participant groups within each of the six activity pattern categories are provided in Table 2; these characteristics are identical to those reported in a previous study (21). Briefly, the analytical and excluded samples were similar except for age, marital status, and educational attainment.” |
|  |  | (b) Indicate number of participants with missing data for each variable of interest |  | N/A |
|  |  | (c) *Cohort study*—Summarise follow-up time (eg, average and total amount) |  |  |
| Outcome data | 15* | *Cohort study*—Report numbers of outcome events or summary measures over time |  |  |
|  |  | *Case-control study—*Report numbers in each exposure category, or summary measures of exposure |  |  |
|  |  | *Cross-sectional study—*Report numbers of outcome events or summary measures | Figure 1 |  |
| Main results | 16 | (*a*) Give unadjusted estimates and, if applicable, confounder-adjusted estimates and their precision (eg, 95% confidence interval). Make clear which confounders were adjusted for and why they were included |  | N/A |
|  |  | (*b*) Report category boundaries when continuous variables were categorized |  | N/A |
|  |  | (*c*) If relevant, consider translating estimates of relative risk into absolute risk for a meaningful time period |  |  |

Continued on next page

| Other analyses | 17 | Report other analyses done—eg analyses of subgroups and interactions, and sensitivity analyses |  | N/a |
| --- | --- | --- | --- | --- |
| Discussion | | | | |
| Key results | 18 | Summarise key results with reference to study objectives | 16 | “This study applied a decision tree approach to understand the interrelated factors associated with working adults’ daily PA patterns and compared the model performance and factor selection to a conventional stepwise multinomial logistic regression approach. Participant’s age (whether older than 60 years of age or younger), the minimum physical strength and minimum skill level required for a person’s job were found to be the most important discriminating factors of daily PA patterns. The decision tree highlighted three distinct homogenous subgroups associated with the “lowest activity pattern”, three subgroups associated with a “moderate activity” pattern, three subgroups associated with the “fluctuating moderate activity” pattern, one subgroup for the “high daytime” and “moderate evening activity” patterns, and four subgroups for the “highest activity” pattern. The factors associated with workers’ PA patterns from the decision tree model included the minimum job skill and job strength levels, sex, educational attainment, fruit/vegetable intake, industry type, work hours, marital status and having a child living at home, computer screen time, and household income group. The CART tree performed similarly to the stepwise logistic regression model with the same input variables. However, the CART approach provided a more parsimonious model with fewer factors than the stepwise approach and showed the hierarchical importance of the selected factors.” |
| Limitations | 19 | Discuss limitations of the study, taking into account sources of potential bias or imprecision. Discuss both direction and magnitude of any potential bias | 20-21 | “a limitation of the SMOTE approach is that it is difficult to determine the number of nearest neighbors, and there is strong blindness in the selection of nearest neighbors for the synthetic examples (35).  The analysis of potential correlates was limited by their availability, for example, information on participants’ time spent outdoors, the quality of the neighbourhood work environment, whether participants worked from home, and behavioural correlates were either not collected in the CHMS or not measured in all survey cycles. While other studies have included current occupation as a potential correlate in decision tree models, our use of the national occupation classification taxonomy to inform ratings of typical physical strengths and job skills required for an occupation provides more detail on the work-related factors (work-related fatigue and job demands-control) that are plausibly related to PA attainment. The CHMS is also a cross-sectional dataset and causality cannot be determined. Furthermore, the CART and stepwise regression approaches include all factors in a single model without explicitly accounting for some factors being causally antecedent or on each others’ causal pathways. The interpretation of continuous variables in the CART methodology is also a limitation, whereby a “split” in a tree is based on arbitrary mathematical conditions which may not reflect widely applied thresholds or recommendations such as the varied cut-offs for fruit/vegetable intake, screen time, and weekly work hours. We contemplated generating random forests, which is a collection of decision trees with each tree fitted from a bootstrap sample of the original data as this may have generated a more accurate prediction model. However, random forests lose the interpretability of individual decision trees (36).” |
| Interpretation | 20 | Give a cautious overall interpretation of results considering objectives, limitations, multiplicity of analyses, results from similar studies, and other relevant evidence | 21 | “Work-related factors, particularly age, job skill level, and physical strength used on the job appeared as the most important discriminators of daily PA patterns, particularly related to differences related to sex, work hours, and industry type. While further improvements of the CART tree model are needed, the approach warrants consideration as a method for identifying the interrelated factors associated with daily PA patterns.” |
| Generalisability | 21 | Discuss the generalisability (external validity) of the study results | 16-17 | “However, the CART approach provided a more parsimonious model with fewer factors than the stepwise approach and showed the hierarchical importance of the selected factors. Accordingly, the CART approach may be a more practical approach compared to convention regression to inform interventions that recognise the complex interrelated factors associated with daily PA in workers, particularly with outcome variables that have three or more categories.” |
| Other information | |  | | |
| Funding | 22 | Give the source of funding and the role of the funders for the present study and, if applicable, for the original study on which the present article is based | 23 | “Funding:  This work was funded by a 2021/22 Data Science to Improve Population Health and Health System Seed Grant (grant number 108702) from the Dalla Lana School of Public Health at the University of Toronto and a project grant from the Canadian Institutes for Health Research (grant number 162244). Funding agencies were not involved in the design of the study and collection, analysis, and interpretation of data and in writing the manuscript.” |

*Give information separately for cases and controls in case-control studies and, if applicable, for exposed and unexposed groups in cohort and cross-sectional studies.

**Note:** An Explanation and Elaboration article discusses each checklist item and gives methodological background and published examples of transparent reporting. The STROBE checklist is best used in conjunction with this article (freely available on the Web sites of PLoS Medicine at http://www.plosmedicine.org/, Annals of Internal Medicine at http://www.annals.org/, and Epidemiology at http://www.epidem.com/). Information on the STROBE Initiative is available at www.strobe-statement.org.
